# Pancreatic Cancer Presenting With Hematemesis and Hemorrhagic Shock Following Anticoagulation With Unfractionated Heparin for Cancer-Related Venous Thromboembolism

**DOI:** 10.7759/cureus.84498

**Published:** 2025-05-20

**Authors:** Mayuko Tagaya, Naoyuki Otani, Ai Sato, Atsushi Irisawa, Yasuhiro Maejima

**Affiliations:** 1 Department of Gastroenterology, Dokkyo Medical University Nikko Medical Center, Nikko, JPN; 2 Department of Cardiology, Dokkyo Medical University Nikko Medical Center, Nikko, JPN; 3 Department of Gastroenterology, Dokkyo Medical University Nikko Medical Center, Mibu, JPN; 4 Department of Cardiovascular Medicine and Nephrology, Dokkyo Medical University Nikko Medical Center, Nikko, JPN

**Keywords:** anticoagulation, cancer-related thrombosis, gastrointestinal bleeding, pulmonary thromboembolism, venous thromboembolism

## Abstract

Cancer-related venous thromboembolism (VTE) is a significant concern owing to its frequent occurrence and status as a leading cause of death in patients with cancer. Cancer-related VTE carries a higher risk of hemorrhage than VTE in patients without carcinoma. A 74-year-old woman with pancreatic head cancer presented with complaints of loss of appetite and weight loss. Contrast-enhanced computed tomography (CT) was performed for stage classification of pancreatic cancer, which incidentally revealed pulmonary thromboembolism. The patient remained clinically asymptomatic, with no evidence of hypoxemia or echocardiographic findings suggestive of pulmonary hypertension. Due to the unavailability of low-molecular-weight heparin for the treatment of acute pulmonary embolism and concerns about potential hemorrhage according to CT findings, unfractionated heparin was selected for its ability to be closely monitored and promptly discontinued if necessary. However, the treatment was immediately halted as the patient developed hematemesis and hemorrhagic shock. Subsequent CT confirmed gastrointestinal hemorrhage, and three-dimensional CT angiography identified the pancreaticoduodenal arcade as the source of bleeding. Emergency catheter angiography and transcatheter arterial embolization were performed, successfully achieving hemostasis. This case highlights the high risk of both recurrent VTE and bleeding complications in patients with cancer-related VTE, underscoring the need for individualized treatment strategies.

## Introduction

Cancer-related mortality has steadily declined, mirrored by a steady increase in the number of cancer survivors. As a result, treatment-related adverse effects have gained prominence [1]. Thromboembolic events during cancer treatment, collectively termed cancer-associated thrombosis (CAT), include both venous thromboembolism (VTE) and arterial thromboembolism. VTE, encompassing deep vein thrombosis and pulmonary embolism, is the second-leading cause of death in patients with malignancies [2,3]. Cancer confers a fourfold higher risk of VTE, with cancer-associated VTE accounting for 30% of all VTE cases [4-6]. In patients with pancreatic cancer, thrombus formation is facilitated by the production of procoagulant substances, such as tissue factor, as well as by vascular stimulation secondary to peritumoral inflammation and tumor progression. Chemotherapy and other anticancer treatments further contribute to this prothrombotic milieu. Conversely, pancreatic tumors typically exhibit poor vascularization, and spontaneous bleeding is relatively uncommon. However, when the tumor invades adjacent structures, such as the gastrointestinal tract, particularly the duodenum, or surrounding vasculature, the risk of clinically significant hemorrhage increases. This risk is further exacerbated in the setting of chemotherapy or radiotherapy-induced bone marrow suppression, which can lead to thrombocytopenia. Patients with cancer-associated venous thrombosis have a higher risk of recurrent thromboembolism and major bleeding during anticoagulant therapy when compared to those without malignancy. These risks are closely associated with the extent of cancer progression [7]. Therefore, careful assessment of these risks is essential to guide treatment strategies.

Clinical practice guidelines from the American Cancer Society (ACS), National Comprehensive Cancer Network (NCCN), and European Society for Medical Oncology (ESMO) recommend low-molecular-weight heparin (LMWH), direct oral anticoagulants (DOACs), and fondaparinux as first-line anticoagulant therapies for CAT. Among these, LMWH and DOACs are most commonly preferred as initial therapy. Unfractionated heparin (UFH) is generally reserved for patients with severe renal impairment. Although LMWH is considered the standard of care globally, it is not approved for use in Japan, thereby limiting its availability. While recent randomized controlled trials have demonstrated that DOACs offer comparable efficacy to LMWH, their use in patients with gastrointestinal malignancies requires caution due to an increased risk of bleeding. Therefore, we chose UFH as an alternative to LMWH. Here, we report the case of a patient with pancreatic cancer and incidental VTE who was started on UFH in the acute phase but unfortunately developed hemorrhagic shock.

## Case presentation

A 74-year-old woman presented to the gastroenterology clinic with a chief complaint of loss of appetite and weight over two weeks. She had undergone abdominal ultrasonography by her previous doctor, who suspected pancreatic head cancer and multiple liver metastases. The patient was referred to our hospital for gastroenterological examination. She was receiving treatment for hypertension but had no history of diabetes mellitus or excessive alcohol consumption. Her vital signs were as follows: blood pressure, 117/56 mmHg; pulse, 91 beats/min; respiratory rate, 16 breaths/min; body temperature, 36.7 ℃; and oxygen saturation (SpO_2_), 98% breathing room air. The results of the blood test are shown in Table 1. 

**Table 1:** Laboratory test results

	Observed value	Reference range
Biochemistry		
Alanine aminotransferase (IU/L)	66	10-42
Aspartic aminotransferase (IU/L)	82	13-30
Gamma glutamyltransferase (IU/L)	568	13-64
Alkaline phosphatase (IU/L)	392	38-113
Total bilirubin (mg/dL)	1.3	0.4-1.5
Total protein (g/L)	7.1	6.6-8.1
Albumin (g/L)	3.4	4.1-5.1
Blood urea nitrogen (mg/dL)	14.9	8-20
Creatinine (mg/dL)	0.7	0.65-1.07
Sodium (mmol/L)	136	138-145
Potassium (mmol/L)	4.0	3.6-4.8
Chloride (mmol/L)	101	101-108
Glucose (mg/dL)	153	73-109
Hemotology		
White blood cell count (×103/L)	11.5	3.3-8.6
Hemoglobin (g/dL)	11.5	13.7-16.8
Platelets (×103/L)	155	158-348
Blood coagulation		
Prothrombin time (seconds)	12.3	11-13.5
Activated partial thromboplastin time (seconds)	28.4	26-40
D-dimer (μg/mL)	39.1	0.0-0.5
Serology		
C-reactive protein (mg/dL)	8.25	0.00-0.14
Carcinoembryonic antigen (ng/mL)	123.2	<2.5
Carbohydrate antigen 19-9 (U/mL)	118,017	<37

**Figure 1:**
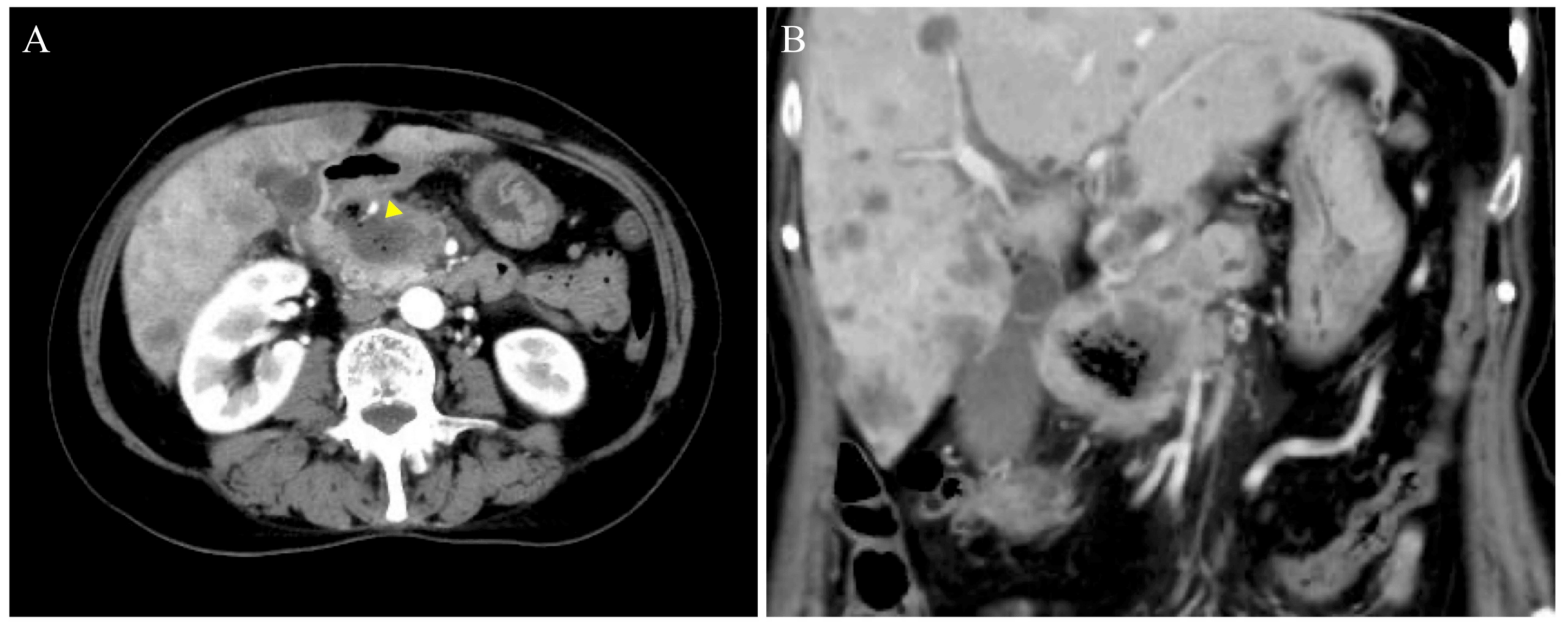
Contrast-enhanced CT of the abdomen Contrast-enhanced computed tomography (CT) of the abdomen revealing pancreatic cancer from the descending duodenal leg to the pancreatic head (A) and duodenal invasion (B). Exposure of the pancreaticoduodenal arcade into the duodenum can be suspected (yellow arrowheads).

**Figure 2:**
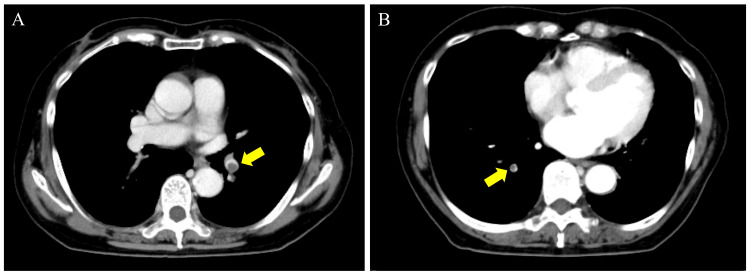
Contrast-enhanced CT of the chest Contrast-enhanced CT of the chest showing thrombi (yellow arrow) in the lower lobe branch of the left pulmonary artery (A) and in the lower lobe branch of the right pulmonary artery (B).

Contrast-enhanced computed tomography (CT) revealed a shadow of internal necrotic tissue extending from the duodenum to the pancreatic head (Fig. 1A). A low-density area of approximately 25 mm with dilatation of the main pancreatic duct was observed in the pancreatic head, and mass invasion reached the descending duodenum (Fig. 1B). Multiple low-density areas of suspected metastases were observed in the liver. The patient was diagnosed with stage IV pancreatic cancer based on the presence of distant metastases. Although the CT scan did not reveal any findings of active bleeding, a pseudoaneurysm arising from the anterior superior pancreaticoduodenal arcade, appearing to erode into the duodenum, was suspected. This anatomical configuration suggested a high risk of hemorrhage due to the direct exposure of the vessel within the gastrointestinal tract. Contrast shadow defects were incidentally detected in the lower lobe branches of both lungs (Fig. 2A, 2B). Bilateral femoral veins showed no shadow defects. The electrocardiogram showed sinus rhythm and no obvious ST-T changes or deep S waves at V5 and V6. Echocardiography revealed no wall motion abnormalities or pulmonary hypertension, with a tricuspid valve flow velocity of 2.8 m/s. The patient was diagnosed with asymptomatic incidental pulmonary thromboembolism.

**Figure 3:**
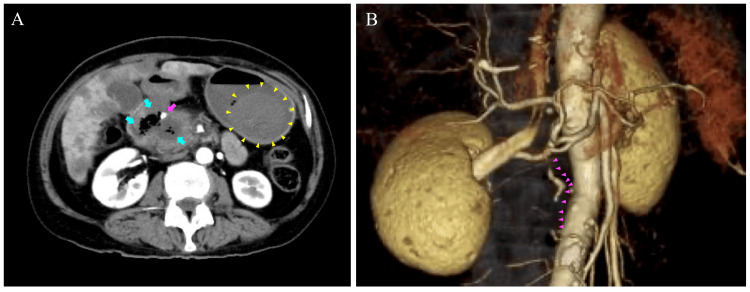
Contrast-enhanced CT and three-dimensional CT angiography immediately after hematemesis The slightly high-density area when compared to the other areas in the gastrointestinal tract on contrast-enhanced computed tomography (CT), immediately after hematemesis, is considered as gastrointestinal hemorrhage or blood clots. The parenchymal border between the duodenum and pancreatic head is indistinct, and intraluminal air is observed within this region (cyan arrows). These findings are suggestive of either duodenal or pancreatic head wall disruption, with possible exposure of the tumor surface into the duodenal lumen. Contrast-enhanced blood vessels can be identified in proximity to the tumor (magenta arrow). No obvious contrast leakage can be observed in the gastrointestinal tract or other areas (A). Three-dimensional CT angiography reveals signs of vasospasm in the pancreatoduodenal arcade, including irregular vessel calibers and filling defects, consistent with post-hemorrhagic vasoconstriction (magenta arrowheads) (B).

Her pulmonary embolism severity index was 104, which was determined to be Class III (moderate) [8]. Hospitalization for anticoagulation therapy was recommended. Given the increased risk of bleeding owing to the exposure of blood vessels into the duodenum, UFH was selected for its ability to be closely monitored and promptly discontinued if necessary. The initial bolus dose of UFH was omitted, and anticoagulation therapy was initiated at a low dose of 4 units/kg/hour. Close monitoring and dose adjustment of anticoagulation therapy were implemented to minimize the risk of hemorrhagic complications. However, the patient suddenly vomited blood 50 minutes after the initiation of UFH. Her consciousness was clear, but her blood pressure dropped to 75/40 mmHg and the hemoglobin level decreased from 11.5 g/dL to 6.5 g/dL. Activated partial thromboplastin time (APTT) measured simultaneously was 30.5 seconds. UFH was discontinued, and rapid fluid resuscitation was promptly administered, restoring her blood pressure to 104/71 mmHg. Since APTT was being monitored and remained unchanged at 30.0 seconds, and her blood pressure was stabilized, the patient did not receive protamine. The hematemesis indicated upper gastrointestinal bleeding. A contrast-enhanced CT was performed again to identify the source of bleeding. The patient had been fasting; therefore, the slightly dense areas noted, when compared to the other areas in the gastrointestinal tract, on contrast-enhanced CT, were unlikely to be food residues and were considered as gastrointestinal hemorrhage or blood clots. There was no obvious contrast leakage in the gastrointestinal tract or other areas (Fig. 3A). 

**Figure 4:**
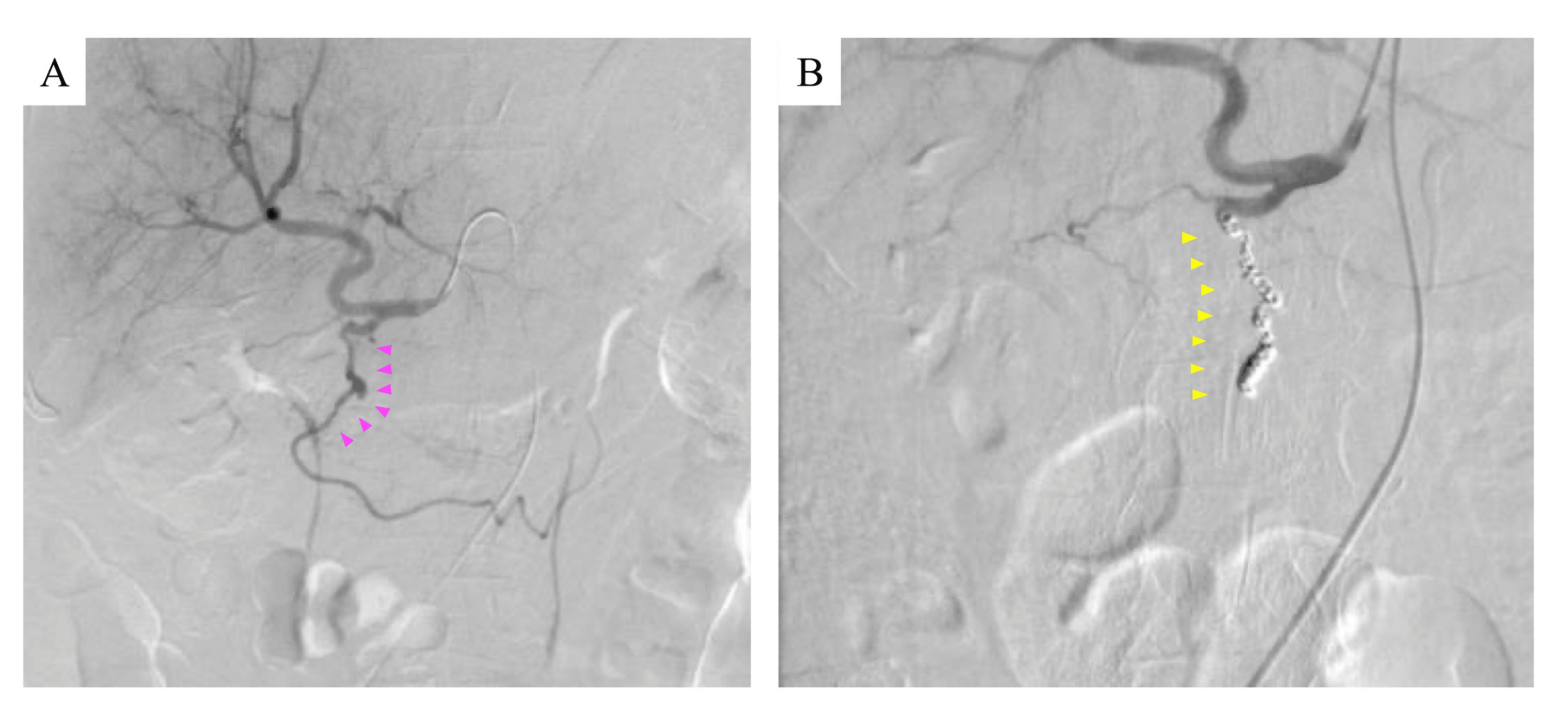
Angiography for pancreaticoduodenal arcade No contrast leakage are noted on pancreaticoduodenal arcade angiography. Pancreaticoduodenal arcade angiography shows arterial spasm (magenta arrowheads) (A). Angiography after transcatheter arterial embolization for pancreaticoduodenal arcade is shown. The loss of blood flow (yellow arrowheads) can be confirmed (B).

Contrast-enhanced CT revealed an indistinct border between the duodenum and pancreatic head, and a low-density area suggestive of intraductal air. These findings suggested the possibility of tumor collapse and subsequent exposure of blood vessels into the duodenum. No evidence of bleeding was found in other organs. Three-dimensional CT angiography revealed signs of vasospasm in the pancreatoduodenal arcade, including irregular vessel calibers and filling defects (Fig. 3B). These findings were consistent with vasoconstriction, which typically occurs following bleeding. We believed that the pancreaticoduodenal arcade within the tumor had ruptured and hemorrhaged into the duodenum. The patient was transferred to another hospital for abdominal angiography and transcatheter arterial embolization of the pancreaticoduodenal arcade, as these procedures were unavailable at our facility. Therefore, transcatheter embolization was subsequently performed using microcoils to cut off blood flow to the gastroduodenal artery (Fig. 4A, 4B). Esophagogastroduodenoscopy (EGD) revealed no active bleeding in the esophagus, stomach, or from the duodenum to the inferior duodenal angulus, and hemostasis was considered successful. An extensive ulcer was noted on the papillary wall of the duodenum, with necrotic tissue present on the surface. The ulcer was considered to be tumor-related, and no exposed blood vessels were identified. A biopsy was not performed due to the tumor’s fragility and the risk of further disintegration. Thereafter, a hemoglobin level of 8.0 mg/dL was maintained after transcatheter embolization, and no progression of anemia was observed. The treatment plan was re-evaluated through discussions among the patient, family members, and healthcare team. It was collectively decided to discontinue the plan involving anticancer drug therapy and transition to palliative care. Based on the EGD findings, the patient and family members were informed of the potential risk of gastrointestinal bleeding from the same site due to tumor progression, as well as the possibility of recurrence or worsening of VTE. Considering these risks, the decision was made to not reinitiate anticoagulant therapy.

## Discussion

In our case, an incidental VTE was found during a close examination of pancreatic cancer. The incidence of VTE varies greatly according to cancer type, with pancreatic cancer having the highest incidence [9]; 8.5% of patients with pancreatic cancer have VTE during cancer diagnosis [10]. Hematogenous metastasis is high in cancer patients with VTE, as the coagulation capacity is enhanced by cancer cells [11]. Our patient had multiple hematogenous liver metastases and had stage 4 pancreatic cancer. Anticoagulation therapy is the standard treatment for VTE for both symptomatic and incidental VTE, as they exhibit similar rates of recurrence and mortality [12]. In Europe and the United States, LMWH is recommended as the standard treatment for cancer-associated VTE. However, in Japan, LMWH is not approved for the treatment of acute pulmonary embolism, thereby limiting its clinical application. Although the use of LMWH was considered for this patient, the time available before administration was insufficient to thoroughly evaluate its appropriateness. DOACs are recommended for managing symptomatic or incidental VTE in cancer patients [1]. Previous studies have shown that DOAC administration is associated with a higher incidence of major bleeding compared to LMWH [13,14]. In the Caravaggio Clinical Trials, apixaban was not inferior to LMWH in the treatment of cancer-related VTE and did not increase the risk of major gastrointestinal bleeding [15]. However, the trial was conducted in the USA, Israel, and European countries, and Asian participants were estimated to be underrepresented. Asian patients are known to be more prone to bleeding complications than patients of other ethnic groups [16]. Anticoagulation with UFH was selected in the present case due to the insufficient evidence supporting the safety of DOAC in Asian patients.

A bolus dose was intentionally omitted due to bleeding concerns, given imaging findings of tumor exposure from the pancreatic head into the duodenum. The initial dose of UFH was set at 4 units/kg/hour, with a plan to start at a low dose, monitor closely, and adjust gradually. Despite this precaution, a bleeding event occurred before the initial scheduled monitoring, rendering proper dose adjustment and monitoring impossible. UFH was immediately discontinued. Given that the bolus dose had been omitted, the infusion rate was low, and APTT was not prolonged, the bleeding was attributed to tumor-related changes rather than the anticoagulant effect of UFH; therefore, protamine was not administered. A contrast-enhanced CT was promptly repeated to identify the source of bleeding. Hematemesis, along with CT findings indicative of gastric hemorrhage, supported the diagnosis of gastrointestinal bleeding. Furthermore, the presence of gastrointestinal air between the duodenum and tumor on CT indicated direct tumor exposure to the duodenal lumen. Three-dimensional CT angiography demonstrated signs of vasospasm in the pancreatoduodenal arcade, including irregular vessel calibers and filling defects, which are characteristic findings following hemorrhage. Based on the anatomical location and imaging findings, we concluded that the source of bleeding was the pancreatoduodenal arcade, likely due to tumor erosion into adjacent vasculature. We hypothesized that tumor invasion and associated pancreatic inflammation had weakened the outer membrane, leading to the formation of a pseudoaneurysm and subsequent bleeding into the duodenum from vulnerable areas of the vessel. Thus, we proceeded with abdominal angiography and transcatheter arterial embolization of the pancreaticoduodenal arcade. Subsequent EGD revealed no active bleeding but showed a tumor with ulceration exposed in the duodenum, along with necrotic tissue at the same site, suggesting fragility. 

Cancer-related VTE is associated with a high rate of major bleeding and VTE recurrence [7]. Furthermore, both recurrence and bleeding are associated with cancer severity. Therefore, the risk of serious bleeding during anticoagulation therapy must be considered in patients with cancer and VTE. The intensity of anticoagulation therapy should be carefully monitored during the first few weeks of therapy, as this is a period associated with a particularly high risk of bleeding. Monitoring anticoagulation intensity during DOAC therapy is difficult, even though andexanet, a neutralizing agent, is available for factor Xa inhibitors. Thus, we used heparin, which can be monitored and promptly discontinued. Assessment scores, such as Registro Informatizado de Enfermedad TromboEmbólica (RIETE) and VTE-BLEED, are used to assess bleeding risk in cancer patients with VTE. The patient had an RIETE bleeding risk score of 2.5, with one point assigned for cancer and 1.5 points for anemia, indicating an intermediate risk, and an estimated incidence of serious bleeding of 2.8% [17]. By contrast, the VTE-BLEED score was 5, calculated based on active cancer (two points), anemia (1.5 points), and age >60 years (1.5 points), categorizing the patient as high-risk, with a bleeding incidence rate of 12.6% [18]. Both the RIETE and VTE-BLEED scores evaluate the bleeding risk during the course of anticoagulant therapy for VTE. Although the RIETE and VTE-BLEED scores share several common variables, the proportions of high-risk patients according to the RIETE and VTE-BLEED scores differ significantly (7.1% and 62.3%, respectively). In addition, both scores demonstrate a suboptimal predictive ability; their positive predictive values remain low, ranging from 0.6 to 3.9, reflecting the relatively low incidence of bleeding [18]. In addition, both scores tend to better predict major extracranial rather than intracranial bleeding. However, it remains unclear whether these scores can predict gastrointestinal bleeding. In the present case, the patient had a high risk of hemorrhage due to tumor-related vascular invasion. Thus, we collaborated with a gastroenterologist to evaluate the tumor location and vascular invasion through imaging studies. Although accurately predicting bleeding events is challenging, we recommend incorporating individualized risk factors, such as imaging findings, into the assessment of gastrointestinal hemorrhage.

## Conclusions

VTE frequently accompanies pancreatic cancer diagnoses, often identified incidentally during imaging. Standard treatment involves anticoagulation therapy, recommended for both symptomatic and incidentally detected VTE. However, our case highlights the critical need to weigh the risk of major bleeding in patients with VTE exhibiting high bleeding risk due to tumor vascular invasion. Current bleeding risk scores, such as RIETE and VTE-BLEED, consider only the presence or absence of cancer and do not account for individualized imaging assessment parameters. Although the true risk of bleeding is difficult to predict, individualized treatment guided by patient-specific bleeding risk assessment is essential.
